# Polyandry provides reproductive and genetic benefits in colonising populations

**DOI:** 10.1002/ece3.6742

**Published:** 2020-09-12

**Authors:** Rebecca C. Lewis, Michael D. Pointer, Lucy A. Friend, Ramakrishnan Vasudeva, James Bemrose, Andreas Sutter, Matthew J. G. Gage, Lewis G. Spurgin

**Affiliations:** ^1^ School of Biological Sciences Norwich Research Park University of East Anglia Norwich UK

**Keywords:** extinction, inbreeding depression, population dynamics, sexual selection, *Tribolium*

## Abstract

Polyandry, when females mate with more than one male, is theorised to play an important role in successful colonisation of new habitats. In addition to possible benefits from sexual selection, even mild polyandry could facilitate colonisation by protecting against inbreeding and reducing the costs of mating with incompatible or infertile males. Here, we measure the importance of mild polyandry for population viability and reproductive fitness following experimental founder events into a higher‐temperature regime. Using colonisation experiments with the model beetle *Tribolium castaneum*, in which females can produce offspring for up to 140 days following a single mating, we founded more than 100 replicate populations using single females that had been given the opportunity to mate with either one or two males and then tracked their subsequent population dynamics. Following population viability and fitness across 10 generations, we found that extinction rates were significantly lower in populations founded by females given polyandrous opportunities to mate with two males (9%) compared to populations founded by monogamous females (34%). In addition, populations founded by females that had been provided with opportunities to store sperm from two different males showed double the median productivity following colonisation compared to monogamous‐founded populations. Notably, we identified short‐term and longer‐term benefits to post‐colonisation populations from double‐mating, with results suggesting that polyandry acts to both protect against mating with incompatible males through the founder event, and reduce inbreeding depression as the colonisation proceeds for 10 generations. Our results therefore show that even mild polyandry provides both reproductive and genetic benefits for colonising populations.

## Graphical abstract

We performed an evolution experiment to test the hypothesis that polyandry can enable populations to successfully colonise new environments. We found that populations founded by polyandrous females were less likely to go extinct and grew larger in size than those founded by monogamous females.

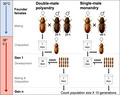

## 1 INTRODUCTION

Populations colonising a new habitat often face an array of challenges, including Allee effects, inbreeding depression, and loss of genetic diversity as a result of small founder population size (Dlugosch & Parker, 2008). Alongside this, colonising populations often need to respond and adapt to a new environment (Sax & Brown, 2000). Understanding the behavioural, ecological, and genetic processes that occur in colonising populations is important for understanding—and ultimately predicting—their establishment probability and is therefore of interest to a range of fields, from pest science to conservation biology (Bouzat, 2010; Fagan, Lewis, Neubert, & van den Driessche, 2002).

One way that populations may be buffered against the challenges posed by colonising new environments is through female multiple mating or polyandry (Candolin & Heuschele, 2008; Parrett & Knell, 2018). In addition to the direct benefits that polyandry may provide to females and their offspring (e.g., increased resources from males) (Fedorka & Mousseau, 2002), multiple mating can provide genetic benefits (Zeh & Zeh, 2001). Polyandry facilitates sexual selection, in which females may encourage genetic benefits for their descendants by skewing paternity towards specific males (Andersson & Iwasa, 1996). Sexual selection can therefore improve population fitness through a range of mechanisms (reviewed in Yasui, 1998), including offspring inheriting “good” or “compatible” genes as a result of polyandry (Neff & Pitcher, 2005) or through selection for increased genetic diversity in offspring, which may increase adaptability to fluctuating or novel environments (García‐González, Yasui, & Evans, 2015).

Polyandry may also provide benefits to individuals and populations in the absence of sexual selection; one recognised mechanism for this is via bet‐hedging (Watson, 1991). If mate‐choice is unreliable or costly, multiple mating may be an effective strategy to protect against unsuitable males. It is still debated whether the fitness gains derived from bet‐hedging are sufficient to drive the evolution of polyandry (Holman, 2016). However, bet‐hedging may play an important role in the dynamics of small colonising populations, where the consequences of mating with a single male who happens to be of low quality or compatibility are expected to be particularly severe (Yasui & Garcia‐Gonzalez, 2016).

Finally, polyandry may play a key role in colonisation through reductions in inbreeding. Cornell and Tregenza (2007) developed a model showing that, because offspring of polyandrous females contain half‐sibs, inbreeding depression in future generations will be significantly reduced by even mild polyandry, improving the probability of colonisation success. This theory received empirical support in a study of seed beetles (*Callosobruchus maculatus*) in which populations founded by polyandrous females had increased fitness after five generations, compared to monogamous females (Power & Holman, 2014). Interestingly, despite the benefits of polyandry for individual fitness, Power and Holman (2014) found no effect of mating treatment on extinction rates, which were low throughout the experiment. It is therefore not yet known how strong or widespread these benefits are across different species and environmental conditions. Given that the strength of inbreeding depression can be dependent on environmental conditions (Armbruster & Reed, 2005), this is an important area for future investigation.

The red flour beetle, *Tribolium castaneum,* is an ideal model system to test experimentally how polyandry influences colonisation success. A pest of stored products, the ecology of *T. castaneum* is characterised by continued colonisation of empty habitats (e.g. grain stores), presumably often by a small number of founders (Dawson, 1977). Females can mate polyandrously and then store sperm to enable offspring production without males for more than 100 days postmating (Michalczyk, Martin, Millard, Emerson, & Gage, 2010). Experimental studies in this species have shown that founder effects have pronounced costs as a result of genetic and demographic effects and that colonising populations are able to rapidly adapt to novel environments (Szucs, Melbourne, Tuff, & Hufbauer, 2014; Szucs, Melbourne, Tuff, Weiss‐Lehman, & Hufbauer, 2017). Further, this species is promiscuous, and experimental evolution studies have shown that a history of strong sexual selection results in decreased risk of extinction under inbreeding and improved invasion into competitor populations (Godwin et al., 2018; Lumley et al., 2015). Moreover, matings and fertility often appear to fail in this species (Tyler & Tregenza, 2013), and there is some evidence to suggest that these costs are reduced when females mate multiply (Pai, Bennett, & Yan, 2005).

Using the *T. castaneum* system, here we test how mild polyandry impacts upon colonisation success when foundresses enter a challenging thermal environment. Following mating opportunities with either one or two males, we placed single females into an empty habitat at 38°C, a temperature which we know is stressful for *T. castaneum* (Dickinson, 2018), and then tracked population dynamics and extinction rates for 10 generations (1 year). We tested the hypotheses that populations founded from polyandrous females (a) were less likely to go extinct and (b) maintained larger sizes due to increased reproductive fitness and then identified the behavioural, ecological, and genetic drivers behind colonisation success. Note that our aim here is not to test explicitly how temperature affects colonisation success, but rather to test how mating patterns affect colonisation success in an environment that is known to be challenging. We discuss our finding in the context of how mating strategy and inbreeding interact to affect subsequent colonisation dynamics.

## 2 MATERIALS AND METHODS

### 2.1 Experimental protocols

All beetles used were from our outbred Krakow Super Strain (KSS), which are reared under standard conditions of 30°C and 60% humidity (Dickinson, 2018). Beetles were maintained both before and throughout the experiment on a fodder medium consisting of 90% organic strong white bread flour mixed with 10% Brewer's yeast and topped with a layer of oats for traction.

**FIGURE 1:**
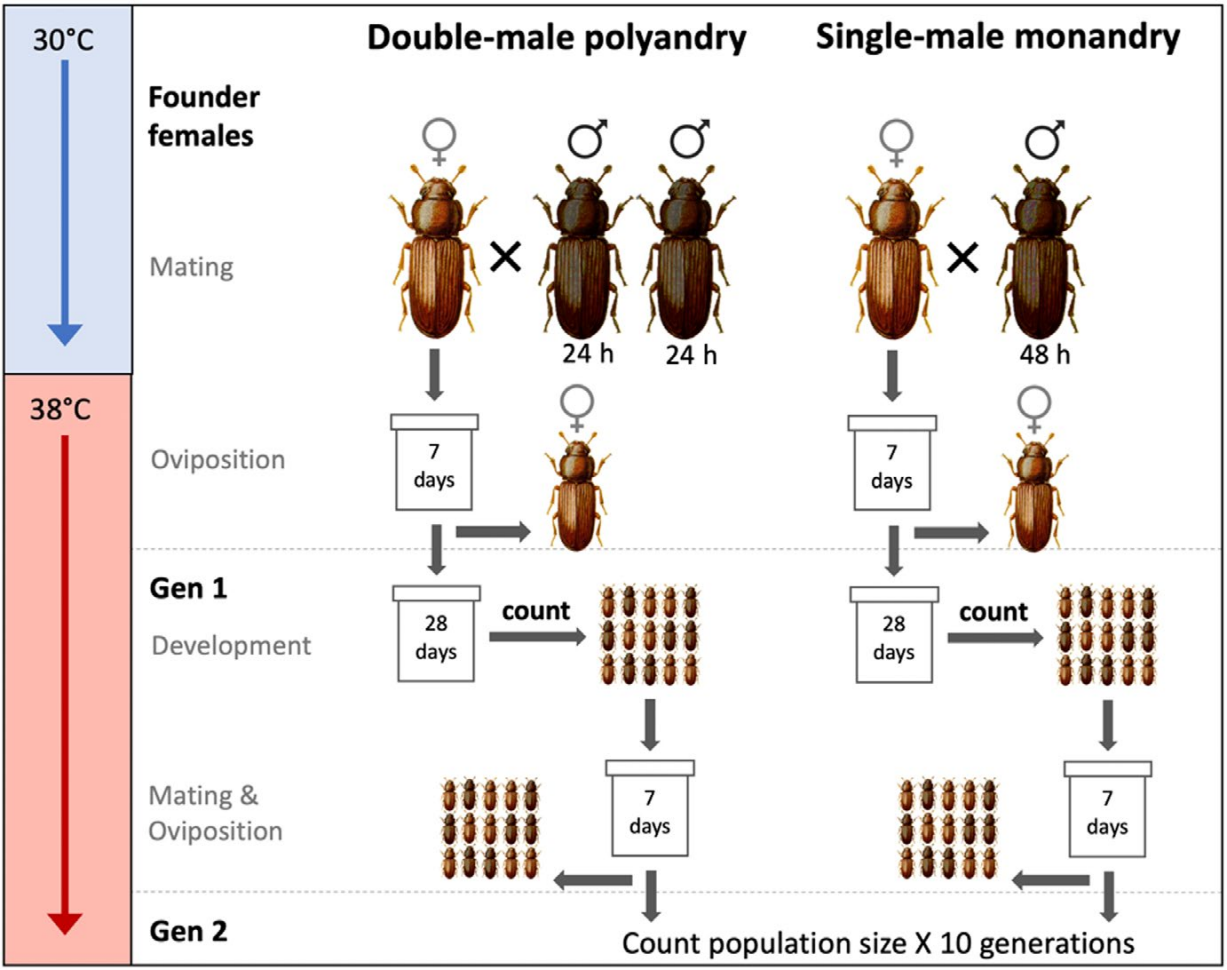
Experimental design for *Tribolium castaneum* colonisations. Individual females were mated with either one or two males, then introduced into a challenging thermal environment to lay eggs. Offspring were counted and used to found subsequent generations. We tracked a total of 108 populations for 10 generations

The overall experimental design is outlined in Figure 1. Founding females and their mates were reared separately and mated under standard conditions as above. To allow matings to occur, pairs were placed into small (7 ml) screw‐top vials containing 1.5 g of fodder. All females received two mating opportunities, each lasting 24 hr. In the first round of pairings, virgin females were randomly paired with virgin males (aged ~7 days posteclosion). In the second round of pairings, half of the females were paired with a second male who had previously been paired with a different female for 24 hr (hereafter referred to as the “polyandrous” treatment). The remaining females were assigned to a “monogamous” treatment, in which they were re‐paired for 24 hr with the same male who, for consistency between treatments, was briefly removed from the dish before being replaced. Thus, all females were paired with single males across two 24‐hr mating periods, either with different males (*N* = 55, polyandry treatment) or with the same male twice (*N* = 53, monogamy treatment). We note that with our experimental design we cannot be sure that all polyandrous females mated twice and that this may result in reduced power to distinguish the true effects of polyandry.

Following the above mating treatments, we then allowed populations to become established in a challenging thermal environment for the remainder of the experiment (Figure 1). Specifically, after 48 hr of mating opportunities with either one or two males, individual females were transferred alone to a population container (100‐ml PVC screw‐cap containers, with the caps pierced for ventilation, containing 70 ml fodder) and allowed to oviposit for 7 days in a warmer thermal regime of 38°C and 60% humidity, after which they were removed and offspring left to develop. This temperature is at the upper limit at which *T. castaneum* can reproduce and presents a stressful and demanding environment for survival and reproduction (Howe, 1960). All population containers postmating were marked only with a randomised ID number so that experimental treatment was unknown by researchers during subsequent handling and counting. Twenty‐eight days after females were removed, the first generation of offspring were separated from the fodder by sieving, the fodder was discarded, and the container and sieve cleaned with ethanol between replicates. The number of live adults was counted and placed into fresh fodder to seed the next generation. If >100 adults were present in a population, 100 were used to seed the next generation, and the remainder discarded after counting (in order to minimise density‐dependent effects). This next new generation of adults was then allowed to mate and oviposit in the fresh fodder for 7 days, after which adults were removed by sieving and the offspring again left to develop into adults for 28 days. This process was repeated for 10 generations, all at 38°C.

### 2.2 Statistical analyses

All analyses were carried out using R version 3.3.3 (R Development Core Team, 2011). We separately modelled how the experimental mating treatment affected (a) the probability of extinction over 10 generations and (b) changes in population size over the same period. For the extinction analysis, we used Cox proportional hazards models, implemented in the Survival package (Therneau, 2020) in R. Because some populations went extinct in the first generation, possibly as a result of failure to mate, fertilise, or develop, we ran the survival models both with and without populations that went extinct in the first generation.

To model how population size changed over time, we used generalised linear mixed models, implemented using the glmmADMB package (Fournier et al., 2012) in R. For this analysis, we only included population counts above zero. Offspring number per generation was modelled as a response variable with a negative binomial error distribution, and generation and experimental treatment (monogamous vs. polyandrous founder) were fitted as explanatory variables, alongside the interaction between treatment and generation. To account for potential nonlinear changes in population size over time, we fitted changes in offspring numbers over generations as (a) a continuous variable and (b) a third‐order polynomial. We fitted random slopes models, which allowed variation among individual populations over generations. Finally, we tested for a difference in population size between experimental treatments in the first and last generations, using two separate generalised linear models (as above but with no random effects), implemented using the MASS package (Venables & Ripley, 2002) in R.

## 3 RESULTS

**FIGURE 2:**
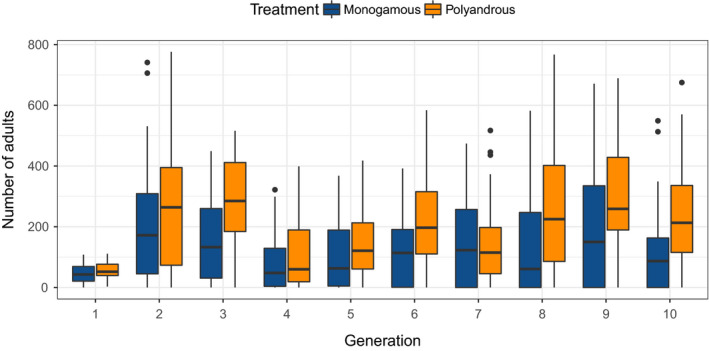
Colonisation dynamics of experimental *Tribolium castaneum* populations founded from monogamous or polyandrous females

We tracked the dynamics of 53 monogamy‐founded and 55 polyandry‐founded *T. castaneum* populations at high temperature for 10 generations or until extinction, with overall dynamics shown in Figure 2. Although there was a general trend for increasing population size postcolonisation, there were substantial fluctuations over some generations, with decreases in population size between generations three and four, and generations six and seven, possibly due to density‐dependent crashes (Figure 2). Despite this variation, we observed a clear and consistent trend for larger adult population sizes in populations founded by polyandrous‐treatment compared to monogamous‐treatment females (Figure 2, tested below). Across all generations, the median size of polyandry‐founded populations founded was 162 (interquartile range = 58–308), compared to 85 (interquartile range = 2–216) for monogamy‐founded populations.

**FIGURE 3:**
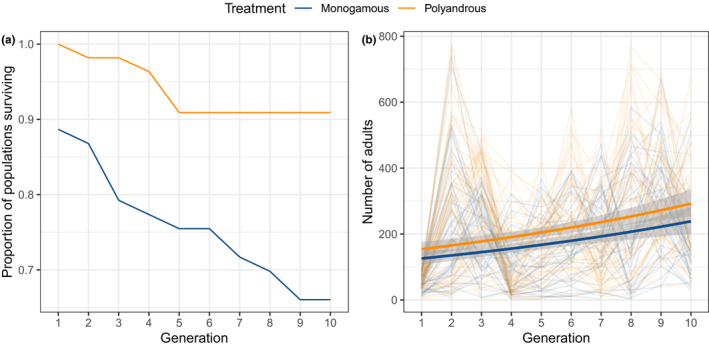
Extinction and population dynamics of experimental *Tribolium castaneum* populations founded from monogamous or polyandrous females. (a) Proportion of populations surviving over time; (b) number of adults in experimental populations. In b, thin lines represent individual populations, while the thick lines represent fitted values from a negative‐binomial GLM

For statistical comparison, we separately tested for differences in extinction rates between mating pattern treatments and population size changes over time. In the first colonisation generation, six populations founded by monogamous females went extinct (11%), while no populations founded by polyandrous females went extinct. By generation 10, 18 monogamous populations (34%) had gone extinct, but only five polyandrous populations (9%) were no longer producing offspring (Figure 3a). The effect of treatment on time to extinction was significant (Cox proportional hazards; hazard ratio = 0.256; 95% CIs = 0.102, 0.642; *p* = .004). This effect remained significant after removal of populations that went extinct in the first generation (hazard ratio = 0.361; 95% CIs = 0.137, 0.950; *p* = .039).

We next tested how founder mating regime affected subsequent population fitness and growth trajectories. Excluding extinctions, we found no significant difference in the number of offspring produced by monogamous or polyandrous females in the first generation (GLM, *p* = .503), suggesting that mating pattern per se did not directly influence offspring production at the initial colonisation event. Considering all generations, however, we found that populations founded from polyandrous females had larger overall population sizes than populations founded by monogamous females (Figure 3b, Table 1). When generation was modelled as a linear continuous variable, population size increased over time, but there was no interaction between treatment and generation (Figure 3b, Table 1). The effect of experimental treatment was also significant when generation was modelled as a third‐order polynomial (*p* = .019). Finally, considering only populations that survived all 10 generations, population size in polyandrous‐founded populations in generation 10 was significantly larger than monogamous‐founded populations (GLM, *p* = .004).

**TABLE 1:** Summary of results from a generalised linear mixed model of population dynamics of experimental *Tribolium castaneum* populations founded from monogamous or polyandrous females. Here, the “treatment” estimate refers to the effect of polyandrous relative to monogamous females, and generation was modelled as a linear effect. As random effects, we modelled a random intercept of population ID (Var = 0.103, *SD* = 0.32) and a random slope of generation with population ID (Var = 0.0008, *SD* = 0.028)

	Estimate	*SE*	*p*
(Intercept)	4.719	0.083	<.001
Treatment	0.229	0.091	.012
Generation	0.063	0.011	<.001
Treatment × Generation	0.007	0.021	.756

## 4 DISCUSSION

Because of the recognised costs to females of mating with multiple males (when a single male can provide full fertility), the widespread evolution and maintenance of polyandry is an evolutionary puzzle (Simmons, 2005). Here, we reveal substantial fitness benefits from polyandry for colonising populations, even when the opportunity for precopulatory sexual selection is experimentally reduced.

Potential indirect benefits of polyandry include: (a) enabling sexual selection, (b) protection via bet‐hedging, and (c) reducing inbreeding load. Perhaps the best‐studied way in which females can increase their fitness through polyandry is via bet‐hedging (Yasui & Garcia‐Gonzalez, 2016). By mating with multiple males, females may reduce the risk of mating failures or being fertilised by an unsuitable male and therefore increase reproductive and/or offspring fitness. Bet‐hedging is likely to be most beneficial when (a) there is a substantial proportion of unsuitable (e.g., infertile) males in the population and (b) the population is small (Yasui & Garcia‐Gonzalez, 2016). In our study, populations were founded by a single female, and as such there is clear potential for polyandry to provide benefits. We found that 11% of monogamous females produced no offspring in the first colonisation generation, while all polyandrous females produced offspring. The low number of extinctions here means that caution is required when interpreting this result. Nonetheless, these percentages are broadly consistent with a situation in which a failure to produce offspring is the result of a failure to mate or due to infertile or incompatible males, from which we can expect only 1.28% of random pairs of males in the double‐mating treatment to both be infertile or incompatible. Previous research in *T. castaneum* has found that a substantial proportion of matings fail to result in offspring production (Pai et al., 2005; Tyler & Tregenza, 2013), and across insects, male infertility or reproductive failure has been observed in the wild (García‐González, 2004). It is therefore likely that multiple mating is one important mechanism for increasing short‐term establishment probability in newly colonised populations through the simple mechanism of gaining successful insemination of functional and compatible spermatozoa.

Another potential mechanism through which polyandry can benefit colonising populations is by reducing levels of inbreeding in subsequent generations (Cornell & Tregenza, 2007). Consistent with this hypothesis, we found significantly lower population sizes and higher extinction rates in monogamy‐founded populations over the full duration of our experiment. Population sizes fluctuated substantially over the course of our experiment, likely a result of density‐dependent processes which are well‐documented in *T. castaneum* (Mertz, 1972). Although the higher population sizes in polyandry‐founded populations were generally consistent over time, it is notable that the difference between treatments was highest when population sizes were high (i.e., in generations 2, 3, 8 and 9) and lowest when population sizes were reduced (i.e., generations 4 and 7). It is possible that a scenario akin to bet‐hedging could explain these longer‐term benefits of polyandry if there was substantial variation in fitness among fertile males, as multiple‐mating would increase the chances of mating with at least one suitable male (Yasui & Garcia‐Gonzalez, 2016). However, this scenario is unlikely to explain our results, as we found no difference in population size between mating pattern treatments in the first colonisation generation, but these became obvious when considering later generations. Similarly, if postcopulatory sexual selection explained some of the differences observed between our experimental treatments, we would expect to observe at least some differences in offspring fitness in the first generation. We therefore suggest that in *T. castaneum* and similar systems, polyandry will benefit colonising populations through two main routes: (a) insuring against male infertility and enabling initial establishment and (b) reducing inbreeding and enabling longer‐term population persistence.

Our results are broadly consistent with a recent study in *C. maculatus*, in which the increased fitness in polyandrous‐founded populations was observed in F4 and F5 generations, but not F1–F3, generations (Power & Holman, 2014). However, and in contrast to our study, Power and Holman (2014) found no effect of mating treatment on extinction, likely because their experimental environment was relatively benign or because there was insufficient time for extinctions to occur. Here, through a longer‐term experiment on colonisation success in a stressful thermal habitat, we demonstrate that the benefits of polyandry persist for longer periods of time and show that they are likely to be important when populations enter challenging environments. Future climate change is expected to result in species shifting their ranges and undergoing changes in population size, and there is increasing realisation that evolutionary processes need to be incorporated into predictive models of population and species responses to climate change (Lavergne, Mouquet, Thuiller, & Ronce, 2010). We recommend that the multiple, interacting benefits of polyandry should be incorporated into such models in order to improve predictive power.

## CONFLICT OF INTEREST

The authors declare no conflict of interest.

## AUTHOR CONTRIBUTIONS

**Rebecca C. Lewis:** Conceptualization (supporting); Investigation (lead); Writing‐original draft (supporting); Writing‐review & editing (equal). **Michael D. Pointer:** Conceptualization (supporting); Investigation (lead); Writing‐original draft (supporting); Writing‐review & editing (equal). **Lucy A. Friend:** Investigation (equal); Writing‐review & editing (equal). **Ramakrishnan Vasudeva:** Investigation (equal); Writing‐review & editing (equal). **James Bemrose:** Investigation (equal); Writing‐review & editing (equal). **Andreas Sutter:** Conceptualization (supporting); Formal analysis (supporting); Methodology (supporting); Visualization (supporting); Writing‐review & editing (equal). **Matthew J. G. Gage:** Conceptualization (equal); Project administration (equal); Supervision (equal); Writing‐original draft (supporting); Writing‐review & editing (equal). **Lewis G. Spurgin:** Conceptualization (equal); Formal analysis (lead); Funding acquisition (lead); Investigation (equal); Project administration (lead); Supervision (lead); Validation (lead); Visualization (lead); Writing‐original draft (lead); Writing‐review & editing (lead).

## Data Availability

The data and code to reproduce all analyses in this manuscript are available on Github (https://github.com/lgs85/TriboliumSexCol).
